# *Toxoplasma gondii* Infection in Pregnant Women: A Seroprevalence and Case-Control Study in Eastern China

**DOI:** 10.1155/2015/170278

**Published:** 2015-10-11

**Authors:** Wei Cong, Xiao-Yan Dong, Qing-Feng Meng, Na Zhou, Xiang-Yang Wang, Si-Yang Huang, Xing-Quan Zhu, Ai-Dong Qian

**Affiliations:** ^1^College of Animal Science and Technology, Jilin Agricultural University, Changchun, Jilin 130118, China; ^2^State Key Laboratory of Veterinary Etiological Biology, Key Laboratory of Veterinary Parasitology of Gansu Province, Lanzhou Veterinary Research Institute, Chinese Academy of Agricultural Sciences, Lanzhou, Gansu 730046, China; ^3^Wendeng Stomatology Hospital, Weihai, Shandong 264200, China; ^4^Jilin Entry-Exit Inspection and Quarantine Bureau, Changchun, Jilin 130118, China; ^5^Affiliated Hospital of Medical College, Qingdao University, Qingdao, Shandong 266071, China; ^6^Wendeng Municipal Hospital, Weihai, Shandong 264400, China

## Abstract

Very limited information is available concerning the epidemiology of* T. gondii* infection in pregnant women in eastern China. Therefore, a case-control study was conducted to estimate the seroprevalence of toxoplasmosis in this population group and to identify risk factors and possible routes of contamination. Serum samples were collected from 965 pregnant women and 965 age-matched nonpregnant control subjects in Qingdao and Weihai between October 2011 and July 2013. These were screened with enzyme linked immunoassays for the presence of anti-*Toxoplasma* IgG and anti-*Toxoplasma* IgM antibodies. 147 (15.2%) pregnant women and 167 (17.3%) control subjects were positive for anti-*T. gondii* IgG antibodies, while 28 (2.9%) pregnant women and 37 (3.8%) controls were positive for anti-*T. gondii* IgM antibodies (*P* = 0.256). There was no significant difference between pregnant women and nonpregnant controls with regard to the seroprevalence of either anti-*T. gondii* IgG or IgM antibodies. Multivariate analysis showed that* T. gondii* infection was associated with location, cats in home, contact with cats and dogs, and exposure to soil. The results indicated that the seroprevalence of* T. gondii* infection in pregnant women is high compared to most other regions of China and other East Asian countries with similar climatic conditions.

## 1. Introduction

*Toxoplasma gondii* is an obligate intracellular protozoan parasite distributed globally in humans and other warm-blooded animals [1, 2]. It is estimated that approximately one-third of the world's human population has been exposed to* T. gondii* [3]. Humans become infected by ingesting food or water contaminated with oocysts shed by cats; by eating undercooked or raw meat containing tissue cysts; or congenitally by transplacental transmission of tachyzoites [1, 4]. Most infections are asymptomatic but in some individuals, especially if immunocompromised, the parasite can become widely disseminated causing severe clinical signs including encephalitis [5]. Primary infection during pregnancy can result in severe damage to the fetus manifested as mental retardation, seizures, blindness, or even death [6]. The rate of congenital transmission and the degree of severity of toxoplasmosis in fetuses vary depending largely on the stage of gestation at the time of infection, the risk of transmission being lower in the first trimester and higher during the last trimester [7]. Therefore, early diagnosis of* T. gondii* infection during pregnancy is very important for prevention of congenital toxoplasmosis.

Epidemiological studies recording prevalence of* T. gondii* infection in pregnant women around the world indicate considerable variation between countries, ranging, for example, from 9% to 67% in European countries [7–12] and reaching as high as 92.5% in Ghana [13]. Similarly, high prevalence of* T. gondii* infection has also been found in some American countries [14–17]. In contrast, prevalence was relatively low in East Asian countries, especially in Korea [18] and Japan [19]. There is little information about the epidemiology of* T. gondii* infection in pregnant women in China [20, 21] and earlier studies have been published in Chinese. This case-control study was performed to estimate the seroprevalence of* T. gondii* infection in pregnant women in two regions of eastern China and to identify associated risk factors and possible routes of contamination.

## 2. Materials and Methods

### 2.1. Study Design

**Figure 1:**
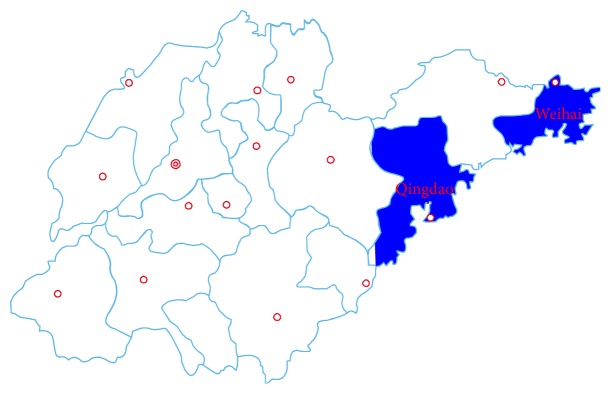
Geographic distribution of study regions in Shandong province, eastern China.

Through a case-control study, we studied the seroprevalence of* T. gondii* infection in pregnant women and control subjects in Qingdao and Weihai, eastern China (Figure 1), from October 2011 to July 2013 and used a questionnaire to identify risk factors and possible routes of transmission.

### 2.2. Sample Collection and Transportation

965 pregnant women attending hospital for antenatal care or medication were recruited for this study. Their age ranged from 17 to 43. A similar number of age-matched nonpregnant women were selected as control subjects. About 5 mL of venous blood was collected aseptically from each of the study participants. Serum separated from whole blood by centrifugation at 2000 ×g for 5 min. was labeled and kept at −20°C until used.

### 2.3. Questionnaire Survey

A structured questionnaire was used to assess risk factors, which included study location, age, residential area, pregnancy status, stage of pregnancy, presence of cats and dogs in home, contact with cats and dogs, consumption of raw/undercooked meat, consumption of raw vegetables and fruits, source of drinking water, and exposure to soil. These variables had been selected based on the literature.

### 2.4. Serological Assay

Sera were tested for anti-*T. gondii* IgG and IgM antibodies using ELISA test kits (Demeditec Diagnostics GmbH, Germany) following the manufacturer's instructions. Optical densities were measured by photometer at a wavelength of 450 nm. Values higher than the cut-off (10 IU/mL) were considered positive. Values ±20% of the cut-off were considered to be equivocal and retested.

### 2.5. Statistical Analysis

Results were analyzed with the SPSS 19.0 software package. For comparison of frequencies among groups, the Mantel-Haenszel Chi square test and, when appropriate, the Fisher exact test were used. Bivariate and multivariate analyses were used to assess the association between characteristics of subjects and* T. gondii* infection. Variables were included in the multivariate analysis if they had a *P* value ≤ 0.25 in the bivariate analysis. Adjusted odds ratios (OR) and 95% confidence intervals were calculated by multivariable analysis using multiple, unconditional, logistic regression. A *P* value < 0.05 was considered statistically significant.

### 2.6. Ethical Considerations

This study was approved before its commencement by the ethical committee of the Affiliated Hospital of the Medical College, Qingdao University, Wendeng Municipal Hospital, and Wendeng Stomatology Hospital. The purpose of and procedures involved in the study were explained and written informed consent was obtained from all participants. Sera were collected with the consent of the volunteers.

## 3. Results

Anti-*T. gondii* IgG antibodies were found in 147 (15.2%) of 965 pregnant women and in 167 (17.3%) of 965 control subjects (*P* = 0.217). Twenty-eight (2.9%) pregnant women and 37 (3.8%) controls were positive for anti-*T. gondii* IgM antibodies (*P* = 0.256). Among pregnant women, 122 (12.6%) were positive for IgG antibodies only compared to 137 (14.2%) of controls. Three (0.3%) pregnant women and seven (0.7%) controls were positive for IgM antibodies only, while 2.6% pregnant women and 3.1% controls were positive for both IgG and IgM antibodies. Detailed information is summarized in Table 1. Univariate analysis of sociodemographic and risk factors for pregnant women and controls identified some factors with a *P* value ≤ 0.25 that may be related to infection (Table 2). Four of these were found to be significantly associated with* T. gondii* infection in multivariable analysis (Table 3): residence area, cats in home, contact with cats and dogs, and exposure to soil.

## 4. Discussion

In this study, we found the seroprevalence of* T. gondii* infection in pregnant women and control subjects to be 15.2% and 17.3%, respectively. These figures are considerably higher than the 7.9% estimated for the general population by a national survey of parasitic disease conducted by the Ministry of Health of China from 2001 to 2004 [22]. However, the prevalence of pregnant women in this study was lower than that observed previously in Liaoning (21.5%) [23], Hubei (22.8%) [24], and Taiwan (31.06%) [25] but much higher than that in Zhejiang (0.17%) [26]. These differences could be related to environmental factors favoring the transmission and infectivity of* T. gondii* oocysts as Qingdao and Weihai both have a marine climate with moist air and abundant rainfall transmission [27], together with an appropriate temperature during most of the year (being neither too hot in summer nor too cold in winter and with an annual average temperature of 12.7°C). Other factors such as different study populations, numbers of cats, diagnostic methods, and living styles may also contribute to these differences.

It is known that felines are the only definitive hosts responsible for contaminating the domestic and wider environment with oocysts. These can remain infective for a long time, especially in water or soil. Contact with domestic cats is often mentioned as a risk factor but there are also contradictory reports. In our study there was a significant association between* T. gondii* infection and the presence of domestic cats in home, indicating that it was a risk factor. This result corroborates with studies reported from France [28] and China [20]. In contrast, some other studies reported an absence of association between* Toxoplasma* infection and the presence of domestic cats in the household [28–30]. Actually, occasional contact with or ownership of cats may not necessarily be a risk factor, whereas frequent exposure to feline feces or neglect of preventive measures (i.e., not washing hands or wearing gloves) may enhance the risk of infection to an appreciable level. In China, with the continuous development of society and improvement of human well-being, more and more people are starting to keep pets, including cats and dogs. This, together with inadequate inspection and quarantine measures, could enhance the potential risk to pet owners of zoonotic hazards such as* Toxoplasma*. Moreover, the prevalence of this parasite among domestic cat populations in different countries may depend on the type of cat (e.g., stray versus pet cats) since stray cats were reported to be more exposed to the parasite than pet cats [31]. In locations surveyed in the present study, large numbers of stray cats roam streets and public places in both urban and rural areas. Not only is it stray cats that pollute the environment indiscriminately but also many owners allow indoor cats to defecate outside home. Consequently, there is a high chance of* T. gondii* oocysts contaminating the environment and being transmitted to humans and it was expected that the prevalence would be higher.

Our data and those of other studies [20, 28, 32] have shown that living in rural or suburban regions with exposure to soil is another risk factor for pregnant women. In some environments, oocysts can remain viable for years [33]. Therefore, all soil, sand, and untreated water should be considered as a potential source of infection for humans; this might explain the higher frequency of infection in pregnant women who frequently come into contact with soil without using gloves compared to women not exposed in this way. In addition, residence is not an isolated factor; in China, rural or suburban residence is generally associated with poorer sanitary facilities, more frequent contact with soil or animals, and drinking unboiled water. These factors enhance the risk of* T. gondii* infection in pregnant women, especially in those who live in rural or suburban regions.

Our results demonstrate that* T. gondii* infection was not associated with consumption of raw vegetables and fruits, consumption of raw/undercooked meat, or specific source of drinking water. Such factors have been found to be significant in previously reported studies [20, 29, 31, 34]. This strongly suggests that, despite our findings,* T. gondii* infection in pigs, cattle, sheep, and chicken in China may nevertheless be contributing risk because local residents frequently consume roasted pork, raw beef, and instant-boiled mutton in the study area. Further large-scale studies should be conducted to estimate the association between risk factors and* T. gondii* infection in China.

It is known that avoiding infection during pregnancy is the most effective method of preventing congenital toxoplasmosis. There are many health centers and obstetric departments, both in China and in the rest of the world, that do not take any measures to prevent or inform patients of the risk of toxoplasmosis. Therefore, the present results serve to alert public health administrative departments of the need to undertake large scale studies to define economic and health impacts of this zoonosis and to formulate guidelines and policies aimed at mitigating its potentially destructive outcomes. In addition, a health promotion strategy in this field should be based on making women of reproductive age aware of infection risk factors, thereby leading to a change in health behavior. Finally, there is also a need to control stray cat populations to reduce the risk of zoonotic transmission of the parasite.

## Figures and Tables

**Table 1:** Combined IgG and IgM anti-*T. gondii* antibodies seroprevalence in pregnant women and controls.

Seroreaction	Pregnant women		Controls		Total		*P* value
	(*N* = 965)		(*N* = 965)		(*N* = 1930)		
	Positive	%	Positive	%	Positive	%	
Positive for IgG only	122	12.6	137	14.2	259	13.4	0.32
Positive for IgM only	3	0.3	7	0.7	10	0.5	0.21
Positive for IgG and IgM	25	2.6	30	3.1	55	2.8	0.49
Negative for IgG and IgM	815	84.5	791	82.0	1606	83.2	0.14
Positive for either IgG or IgM	150	15.5	174	18.0	324	16.8	0.14

**Table 2:** Sociodemographic and risk factors associated with *Toxoplasma* seropositivity in pregnant women and controls by univariate analysis.

Characteristic	Pregnant women (*N* = 965)				Controls (*N* = 965)				Total (*N* = 1930)			
	Number tested	Number positive	%	*P* value	Number tested	Number positive	%	*P* value	Number tested	Number positive	%	*P* value
Age group (years)												
25 or less	339	47	13.9	0.17	252	44	17.5	0.49	591	91	15.4	0.03
26–35	467	68	13.7		551	90	16.3		1018	158	15.5	
>35	159	32	20.1		162	33	20.4		321	65	20.2	
Location												
Qingdao	445	69	15.5	0.83	415	77	18.6	0.37	860	146	17.0	0.45
Weihai	520	78	15.0		550	90	16.4		1070	168	15.7	
Residence area												
Urban	534	70	13.1	0.04	386	60	15.5	0.24	920	130	14.1	0.02
Rural	431	77	17.9		579	107	18.5		1010	184	18.2	
Cat at home												
Yes	14	4	28.6	0.16	108	33	30.6	<0.001	122	37	30.3	<0.001
No	951	143	15.0		857	134	15.6		1808	277	15.3	
Dog at home												
Yes	182	22	12.1	0.19	280	35	12.5	0.01	462	57	12.3	0.009
No	783	125	16.0		685	132	19.3		1468	257	17.5	
Contact with cat and dog												
Yes	203	74	36.5	<0.001	429	113	26.3	<0.001	632	187	29.6	<0.001
No	762	73	9.58		536	54	10.1		1298	127	9.78	
Consumption of raw vegetables and fruits												
Yes	545	92	16.9	0.11	688	127	18.5	0.14	1233	219	17.8	0.02
No	420	55	13.1		277	40	14.4		697	95	13.6	
Consumption of raw/undercooked meat												
Yes	692	108	15.6	0.61	625	118	18.9	0.08	1317	226	17.2	0.12
No	273	39	14.3		340	49	14.4		613	88	14.4	
Exposure to soil												
Yes	572	98	17.1	0.048	731	137	18.7	0.037	1303	235	18.0	0.002
No	393	49	12.5		234	30	12.8		627	79	12.6	
Source of drinking water												
Tap	715	110	15.4	0.83	642	112	17.4	0.871	1357	222	16.4	0.87
Well + river	250	37	14.8		323	55	17.0		573	92	16.1	

**Table 3:** Multivariate analysis of selected characteristics of the participants and their association with *T. gondii* infection.

Characteristic^a^	Adjusted odds ratio^b^	95% confidence interval	*P* value
Residence area	1.55	1.15–2.18	0.010
Cats in home	3.45	2.40–4.91	<0.001
Dogs in home	1.08	0.87–1.49	0.48
Contact with cats and dogs	3.07	2.33–4.12	<0.001
Consumption of raw vegetables and fruits	1.03	0.81–1.31	0.64
Consumption of raw/undercooked meat	1.13	0.83–1.53	0.44
Exposure to soil	1.66	1.18–2.34	0.004

^a^The variables included were those with a *P* < 0.25 obtained in the bivariate analysis.

^b^Adjusted by age and the rest of characteristics included in this table.
